# Should We Treat Depression with drugs or psychological interventions? A Reply to Ioannidis

**DOI:** 10.1186/1747-5341-6-8

**Published:** 2011-05-10

**Authors:** John M Davis, William J Giakas, Jie Qu, Pavan Prasad, Stefan Leucht

**Affiliations:** 1 Gilman Professor of Psychiatry, Psychiatric Institute, Department of Psychiatry, University of Illinois at Chicago and University of Maryland Psychiatric Research Center, Chicago, IL, and Baltimore, MD, USA. 1601 W. Taylor Street, 508W, Chicago, IL, USA; 2 Rockford Psychiatric Medical Services, S.C., Rockford, IL 61107 USA; 3 Department of Psychology-Neuroscience Track, Yale University, New Haven, CT, USA; 4 Psychiatric Institute, Department of Psychiatry, University of Illinois at Chicago USA; 5 Klinik fur Psychiatrie und Psychotherapie der TU-Munchen. Ismaningerstr. 22, 81675 Munchen, Germany

## Abstract

We reply to the Ioannidis's paper "Effectiveness of antidepressants; an evidence based myth constructed from a thousand controlled trials." We disagree that antidepressants have no greater efficacy than placebo. We present the efficacy from hundreds of trials in terms of the percentage of patients with a substantial clinical response (a 50% improvement or more symptomatic reduction). This meta-analysis finds that 42-70% of depressed patients improve with drug and 21%-39% improve with placebo. The response benefit of antidepressant treatment is 33%-11% greater than placebo. Ioannidis argues that it would be vanishingly smaller because systematic biasing in these clinical trials would reduce the drug-placebo difference to zero. Ioannidis' argument that antidepressants have no benefit is eroded by his failures of logic because he does not present any evidence that there are a large number of studies where placebo is substantially more effective than drug. (To reduce to zero, one would also have to show that some of the unpublished studies find placebo better than drug and have substantial systematic or methodological bias). We also present the empirical evidence showing that these methodological concerns generally have the opposite effect of what Ioannidis argues, supporting our contention that the measured efficacy of antidepressants likely underestimates true efficacy.

Our most important criticism is Ioannidis’ basic underlying argument about antidepressants that if the existing evidence is imperfect and methods can be criticized, then this proves that antidepressant are not efficacious. He presents no credible evidence that antidepressants have zero effect size. Valid arguments can point out difficulties with the data but do not prove that a given drug had no efficacy. Indeed better evidence might prove it was more efficacious that originally found.

We find no empirical or ethical reason why psychiatrists should not try to help depressed patients with drugs and/or with psychotherapeutic/behavioral treatments given evidence of efficacy even though our treatment knowledge has limitations. The immense suffering of patients with major depression leads to ethical, moral, professional and legal obligations to treat patients with the best available tools at our disposal, while diligently and actively monitoring for adverse effects and actively revising treatment components as necessary.

## 

We were asked to write a reply to the Ioannidis's paper "Effectiveness of antidepressants; an evidence based myth constructed from a thousand controlled trials," a critique extended to behavioral and psychotherapies, as well [1]. However, after agreeing to this task, we realized that the paper was an excellently written paper and that we agreed with most of Ioannidis's points and except one point: his statement that both antidepressants and psychotherapeutic/behavioral treatments have no efficacy. A brief summary of Ioannidis's paper is as follows:

Ioannidis says that antidepressant use is based on "pseudo-evidence-based medicine" and that there is "no reason to take antidepressants." He characterizes antidepressant effectiveness as a "myth" that is misleading the public into believing that this class of medicine is useful, and he builds his argument along several lines. Ioannidis quotes evidence that drug companies fail to publish some clinical trials, often trials where their drugs are not found to be as effective as they desire. He argues that the effect size of antidepressants is small, and would be even smaller if many negative trials were published. He describes methodological problems with clinical trials of antidepressants (see below), which he believes would also reduce the true effect size, and concludes that "the difference became large enough to be clinically important only in the very small minority of patient populations with severe major depression." He further claims that although antidepressants appear to show better efficacy with increasing severity of depression in some studies, he views this as attributable to placebos having decreased efficacy, rather than antidepressants being more effective by comparison.

Ioannidis argues that antidepressant have essentially no efficacy because:

(Point 1) The drug companies suppress negative studies, biasing the literature.

(Point 2) Drug-placebo differences are small, and presumably vanish because of this suppressed evidence and because of methodological imperfections in clinical trials:

i) Studies have outcomes that are "non-relevant outcomes", too small an improvement to be clinically relevant.

ii) Studies are too short.

iii) The statistics used to analyze data falsely exaggerate drug-placebo differences.

iv) Using too many exclusion criteria might inflate drug-placebo differences.

v) Placebo lead-in periods falsely inflate the drug-placebo differences.

vi) The use of multiple active treatment groups (several groups versus one placebo group) is unethical and might reduce the drug-placebo difference.

(Point 3) The drug-placebo difference is larger in the more severely depressed subgroups and in the older studies, which included more severely depressed patients, so for the majority of patients the effect size must be negligible.

(Point 4) Since the cause of depression is complex and multifactorial with both biological and non-biological etiology, you would not expect a large antidepressant effect size.

(Point 5) Antidepressants have unknown risks.

(Point 6) Depression is over-medicalized and over-treated.

Ioannidis sometimes phrases his conclusions as conditional statements: "if most of the antidepressants efficacy is simply the placebo effect...." or "these agents may be of clinical use only in severely depressed people." These and other statements plus the title that antidepressant efficacy is a myth all suggest that Ioannidis is asserting that antidepressants do not help depressed patients at all except for his hedge, that it may help "a very small minority." He then states some of the implications of this lack of efficacy.

We begin by (a) describing our five points of agreement with Ioannidis, (b) explaining what depression is in humanistic perspective, (c) reviewing the evidence on efficacy, and Ioannidis's critique, (d) discussing Ioannidis's implications, and (e) placing the issue of antidepressant treatment in a philosophical and ethical perspective.

## While we agree with five of the points Ioannidis raises, we disagree with point 2

### Five Points of Agreement

#### (Point 1) Drug companies suppress negative studies

We agree with Ioannidis that suppression of clinical trials through failure to publish is not good science. We believe that this is as fraudulent to medicine as Enron, Lehmann, and Madoff are to finance. (Of course there are shades of grey here.) Trials on many medical, non-psychiatric, drugs are suppressed [2] and antidepressants are no exception. The burden of proof is on the placebo-controlled study because it must be a good enough study to capture real efficacy differences over error. The regulatory issues are too complex to discuss here, such as the use of large samples to achieve statistical significance of a small effect size, but regulatory issues are far different than issues regarding real-life clinical treatment choices. All controlled studies should be published, including failed studies (a failed study is where the established drug comparator was not found to be better than placebo) in order to provide a complete and comprehensive accounting of the drug, and because they contain other useful information (e.g. what dose is too low a dose for full efficacy). We disagree with one drug company's response to the Turner article [3], justifying their failure to publish because of "failed trials." If one drug company suppresses studies where their drug is compared against another, it may create an illusion that their new drug is more efficacious and safer than they actually found, obscuring drug differences.

#### (Point 3)

The drug-placebo difference is larger in the more severely depressed subgroups and in the older studies, which included more severely depressed patients.

We agree.

#### (Point 4) The cause of depression is complex

We agree that biological factors do not explain the entire story in Major Depressive Disorder. One of us was among the three NIMH psychiatrists who originally formulated the idea that depression could be an imbalance of biogenic amines, but none of us at that time believed it was the sole cause, and stated in our original papers [4] that psychosocial factors are important. Furthermore, there may have been many different types of biological, environmental, causes (e.g. dietary), epigenetics, and different genomic factors. Some biological subtypes may not be helped by current antidepressants. Consequently, we agree that a complete cure with antidepressants alone would not be expected.

#### (Point 5) Antidepressant treatment has risk

We agree with Ioannidis that antidepressants cause both known and unknown adverse effects, and both risks and benefits must be balanced. We also acknowledge that there have been concerns regarding whether certain antidepressants may cause suicides.

#### (Point 6) Over-medicalization

We do agree that there is a problem of over-medicalization in the treatment setting, where patients and physicians sometimes look for a "quick fix." Antibiotics are widely given when they should not be, but this does not prove antibiotics are not useful. Over-use or inappropriate use is common to many treatments and more usefully discussed as such. When presented with a depressed patient, physicians want to do something, both because of the physician's desire to help and because of the patient's expectation to receive tangible treatment (i.e. a prescription). Therefore, a prescription can sometimes be almost a reflex, without always considering the full benefits, risks, and alternatives. However, there is evidence that most depressed patients are not treated at all or are under-treated, and more are receiving psychotherapy without medications than medication without psychotherapy. Ioannidis does not recommend either or both. Most psychiatrists would not recommend initial drug treatment for a person who is simply discouraged. Finally, direct to consumer advertising may lead to over-medicalization. We would favor legislation prohibiting direct advertisements to patients, limiting the amount of money spent on promotion of medication, and structuring the pharmaceutical industry, so as to provide more incentive for developing new medications and less incentive for selling a costly variant on an old medication, offering minimal clinical advantage through wide-spread advertisement. It is true that antidepressants are used for many conditions, other than depression, and some have proven efficacious (e.g. for panic attack disease, obsessive compulsive disease, etc.). Many medical drugs are serendipitously found to help entirely different diseases, accountings for almost 50% of the indications for drug treatment. We do not disagree with many of Ioannidis's legitimate concerns, which are widely applicable over all of science and medicine.

## Humanistic Significance of Major Depressive Disorder

Major Depression is a mental disorder that can have a devastating impact on the individual suffering from the condition as well as on those closest to them, such as family, friends, and colleagues who are affected by the individual's loss of function and emotional absence. Major Depression is not simply a state of transient sadness, but it is also characterized by persistent mental dysfunction that affects mood, cognition, behavior, and function--every facet of a person's existence. Depression can be triggered by external events or may occur independently without obvious environmental stress or precipitants. Individuals who suffer from depression, first and foremost, lose the pleasure and enjoyment that make life worth living and believe their situation is unalterable [5]. Life may become bleak, empty, or pointless and the individual can experience despair, tearfulness, anxiety, agitation, appetite changes, loss of energy, loss of motivation, sleep disturbances, impaired concentration and attention, low self-esteem, feelings of worthlessness, excessive worry, irritability, temper outbursts, decreased interest in activities including sex, excessive guilt, painful physical feelings, and suicidal feelings and behaviors, including actual suicide. The most severe cases can develop psychotic symptoms, including delusional ideation and hallucinations. Intense suffering and physical pain rarely lead to suicide in normal individuals, but the suffering in depression is so intense that suicide is common, the life-time prevalence being 5-15% before biological treatments were discovered [6-8]. The World Health Organization (WHO) [9]states that "Almost one million people commit suicide every year," and that "Depression is ranked as the leading cause of disability worldwide and affects around 120 million people worldwide."

Those who have had a single episode have a 60% chance of having a second episode and a 90% chance of recurrence after a third episode, and some patients can experience many more episodes [10]. Those who commit suicide leave behind spouses, children, and friends who must deal with the aftermath of the tragedy. In a large population-based Danish study, using the extensive data from the national registry, increased use of antidepressants was associated with a 10% decline in suicide rates.

Depression is a disease that not only brings a heavy personal toll, but has a substantial economic cost to families and to society as well. It is unrealistic to believe that someone can be "talked out of" depression or that they should simply "pull themselves up by their bootstraps." That would be tantamount to telling an acute asthma patient that they should "will themselves" to stop their asthma attack. While there is much we do not know about depression, there is mounting evidence that it has both hereditary loading and environmental risk factors. Persistent, untreated depression produces a type of neurodegenerative disorder, associated with synaptic changes [11,12], and alterations in brain region metabolism [13,14] among other findings. Similar to poor control of blood sugar in diabetics, poor control of symptoms in Major Depression is associated with worse long-term outcome and greater overall disability.

When faced with a sick patient, clinicians choose the best available option for that patient, factoring into the decision all aspects unique to that particular individual (i.e. personality, cognitive ability, other medical illness, substance use, prior history, risk of self-harm, family dynamics, etc.). Certainly, controlled trials should be considered, but the physician or therapist knows much more about the patient than just the percent response of symptomatic volunteers, including knowing the patient's overall severity, past history of response to previous treatment, symptom constellation, psychological and environmental factors, family history and the risks of treatment. Should available therapies not be used because they do not meet an unattainable standard of evidence? Should we postpone treatment until we have the perfect therapy? The failure to treat has consequences. Most surgical treatments are not proven by double-blind studies and can, therefore, be criticized. There are treatments that should not be used, even if the patient wants them. But, does Ioannidis offer sufficient rigorous evidence that antidepressants are one of these?

## Are Antidepressants Efficacious?

The primary clinical treatment efficacy outcome markers are response, remission, and relapse. Response is the degree to which an antidepressant or placebo reduces the severity of the depression. The definition of response in most antidepressant studies is a 50% symptom reduction. Remission is considered as a full response, with absence of depressive symptomatology. Some patients have some degree of response and do not become fully remitted. Response and remission are generally parallel. Relapse, however, is when a patient improves and later develops another depressive episode.

Physicians initiate a plan of care and monitor patients for evidence of reduction of symptoms (response). Efforts to optimize treatment outcome to achieve a full remission are important because of the increased risk of relapse in those patients who only achieve partial remission. Patients who achieve partial response or partial remission are more likely to relapse and have greater mortality and morbidity. Antidepressants are not a cure. Many patients are not fully remitted. Almost all of the patients will relapse once they stop taking the medication and some have residual symptoms.

Ioannidis presents very little actual data to support his conclusion that antidepressants are not efficacious, which is based essentially on one effect size from one study (Turner) [3]. This study is based primarily on data from mildly depressed patients and non-patient volunteers for a few antidepressants of the many ever studied, with an effect size of 0.33. Ioannidis does quote several other meta-analyses without actually stating the effect sizes of other antidepressants. He argues that because some studies are not published by the drug industry, the measure of effect size would actually be less. He does a simulation of how big this effect could be. Though simulations have value, they can be wrong. An example of such a simulation is the risk models used by the financial industry, which calculated that there was no significant risk of mortgages based securities defaulting. Nevertheless, mortgage defaults were one of the important causes of the meltdown of the financial systems in the last several years. Since Ioannidis bases most of his argument on this study of Turner [3] and Kirsch [15], we note that a review of antidepressant trials submitted to the pharmaceutical industry to the European Regulatory Authority found [16] that only one study was not reported. The problem of negative studies may not be as severe as Ioannidis suggests. Fountoulakis and Moller [17] have recalculated the effects size reported by Kirsch, finding a greater drug effect than that stated by Kirsch [15].

While he states there were a thousand random controlled studies, Ioannidis avoids discussing actual data. As these meta-analyses occurred recently, Ioannidis could not know of the findings, and this calls into question the data on which Ioannidis based his conclusion. Ioannidis states (and we agree), that antidepressants, on average, have similar efficacy (although there are minor differences) [18], therefore, evidence on all these drugs can be considered en mass. Since there is a large body of placebo-controlled randomized studies, we also examine the evidence, from a wide range of antidepressants, using meta-analysis that reviews hundreds of studies. Meta-analysis of antidepressant trials of the 1950s-1990s focusing on the tricyclic antidepressants and monoamine oxidize inhibitors, which includes studies of more severe, hospitalized, or suicidal patients, found about 61-70% of patients responding to drug and 27-35% to placebo (e.g., a drug-placebo difference of about 30%) [19-21] (See Table 1). We also present the ratio of the percent improvement of drug divided by the percent improvement of placebo, which is roughly equivalent to risk ratio. A ratio of 2 indicates that antidepressants show twice the rate of improvement of placebo. We will report percent improvement in this paper, since it is simple to understand. (A problem with statistical parameters is that the general reader may not understand the statistics and must rely on the spin the author puts on it. A simple percent facilitates the reader in forming a gestalt of the data.) Many of the early trials were done by academic clinicians, some supported by granting agencies such as the NIMH [22-25] or British Medical Research, finding essentially the same drug-placebo differences as industry, and where there was no commercial motive not to publish negative studies. We agree with Ioannidis that drug-placebo difference has decreased in recent years [26,27]. He supports this assertion, quoting Barbui [28], concluding that "there is "absolutely" no difference between paroxetine and placebo," but this study actually reported significant efficacy, finding that 52% of drug-treated patients improved compared to 40% of placebo patients, a typical finding more characteristic of recent studies of mild depression and symptomatic volunteers. Barbui was referring to all-cause *discontinuation*, an outcome which does not measure efficacy per se, but rather an outcome more of a measure of side effects than of efficacy. The similar all-cause discontinuation rates found in this study reflects that dropout due to medication side effects is about equal to dropouts from placebo due to poor efficacy. The older studies have greater effect sizes than the newer studies and included more moderately and severely ill patients. Due to changes in defining symptom severity over the years, "mild" or "severe" are relative terms, and a mildly ill patient in an earlier study might be considered a severely ill patient in a more recent study. There are, therefore, many differences between early and later studies. We make no claim that any meta-analysis reflects the true size effect of antidepressants. Our argument is that a low effect size estimate is clinically significant to some patients, and that the effect size, the percentage difference of drug and placebo, is not zero.

**Table 1:** Response to antidepressants in the treatment of an episode.

	Percent Response			Drug/Placebo	
**Initial Antidepressant Studies 1957 ~1990**:	**Drug**	**Placebo**	**Difference**	**Ratio**	**References**

Very early 1957-1974 Imipramine	70%	39%	31%	1.8	[19]

1953-1990 All older antidepressant (TCA class)	63%	36%	27%	1.7	[20]

1953-1990 All older antidepressant (MAO class)	66%	32%	35%	2.1	[20]

1973-1980 Trazodone	61%	29%	32%	2.1	[21]

Early fluoxetine (Prozac) studies	64%	32%	32%	2.0	[77]

More Recent Studies to Present:					

Antidepressant (TCA's ~1979-91))	46%	31%	15%	1.5	[78]

Venlafaxine (a newer antidepressant)	45%	25%	20%	1.8	[79]

Severe outpatient depression (Duloxetine, a newer antidepressant)	42%	21%	21%	2.0	[35]

Duloxetine (all patients)	48%	35%	12%	1.3	[35]

Paroxetine (a newer antidepressant) Ioannidis example of no efficacy	53%	43%	11%	1.2	[28]

The table summarizes meta-analyses of antidepressant efficacy including both studies done from 1957 up until the 1980s or 1990s and studies done from roughly the 1990s to the present. There is excellent agreement that all the antidepressants have roughly the same efficacy, although there may be some minor differences [18]. We would caution against making causative inferences between the earlier and the later studies, as cause should not be attributed to inferences from correlational or observational comparison, though they can support inferences. The study Ioannidis cites is in the bottom row.

## Prevention of Future Episodes

An important antidepressant effect is the prevention of future episodes in people with recurrent episodes. The first author found (in the first meta-analysis done in psychiatry) that antidepressants prevent relapses, in that 53% of the placebo patients relapsed, whereas only 27% of drug-treated patients relapsed [29], showing that drugs decrease the relapse rate to less than one-half of the placebo rate. Also, the more recent meta-analysis that included only new antidepressants confirmed this finding [30-32], and one of these was cited by Ioannidis [18]. Most of these studies were 6 month to 2 years. An NIMH study found a good preventative effect in the first 3 years (65% placebo relapsing and 14% on drug relapsing) [22]. It is true that most of these studies were 2 years or less, but a 4-5 year extension of this study, in which the patients who had not relapsed on drug at the end of 3 years were then randomized, found that 67% on placebo relapsed and 10% on drug relapsed in the next two years [22,23,33]. A meta-analysis of trials of 6, 12, 18, 24, 36 months found the reduction of relapse rate to be essentially the same for all durations, and the 36 month duration group had a relapse rate of 72% on placebo versus 30% on antidepressants [34]. There is no indication that the relapse rate from antidepressant therapy increases with the duration of treatment. In other words, antidepressants provide effective prophylaxis against relapses. Since the drug either prevents or delays relapse, patients will spend less of their lifetime in a depressive episode. Long-term morbidity and disability can be diminished by treatment. Thus there are two components to treatment. Patients may recover sooner with treatment and also have fewer (over 50% less) recurrences than without treatment. We provide an example to illustrate the cumulative effect of shortening episodes and prevention of future episodes: An untreated, unipolar, non-psychotic, severely depressed patient may have 10 episodes during a lifetime, each lasting 2 years and could spend a total of 20 years in depressed state (10 episodes each 2 years). With drug treatment, the same patient could have only 5 episodes (the preventative effect), each lasting a month (the effect of treating an episode), resulting in a total of 5 months lifetime depression. This example, of course, implicates that the antidepressant effect does not fade away over the years.

## Ioannidis' Criticism of Antidepressant Trials

Ioannidis labels the effect size he reported as small, and we will not argue about the label, "small". We present data in Tables 1 and 2 based on a range of studies of the most widely used antidepressants. This includes hundreds of placebo-controlled randomize double-blinded trials (the best controlled of studies in the evidence hierarchy), conducted throughout the world, by scientists in industry as well as in academic settings, but we cannot cover all of Ioannidis's 1000 randomized controlled trials. The efficacy is consistent with pragmatic, epidemiological, service research and clinical research. Note the agreement between the studies Ioannidis quotes and ours for the newer studies, as summarized in Tables 1 and 2. His argument is that the effect size would be reduced to essentially zero (i.e. antidepressants are not materially superior to placebo since the data is based on biases from the suppression of negative studies by industry and smaller still if you counted in negative unpublished studies); and the 6 biases, which would surely decrease it further. We question the logic of Ioannidis' assertion that industry bias could reduce efficacy to zero. To do so, there would have to be an equal number of studies showing drug worse than placebo, and Ioannidis fails to show any evidence that this is the case. He starts with an effect size of 0.31 and assumes suppression of negative studies would reduce it still more, but his effect size of 0.31 is based on all studies in this FDA report-not just published study. As industry is required by law to report all studies to FDA for registration, this is another reason to doubt his premise.

**Table 2:** summarizes the percent of patients relapsing on placebo or drug in several meta-analyses and from one individual NIMH supported 5-year study.

	Percent Relapse				
**Maintenance Studies**	**Drug**	**Placebo**	**Difference**	**Ratio/drug**	**Reference**

Early 1953-1976	27%	53%	-26%	2.0	[29]

Three Year Treatment imipramine	30%	72%	-47%	3.4	[22,23,33]

Imipramine group from 3 year study randomized to drug or placebo yr. 4-5.	10%	67%	-57%	6.7	[22,23,33]

Later 1953-2003	18%	41%	-23%	2.3	[30]

Later 1998-2006	23%	50%	-27%	2.2	[32]

Ioannidis states, and we agree, that current evidence suggests that more severely depressed patients show a larger absolute degree of improvement relative to placebo controls than do more mildly depressed patients and symptomatic volunteers [35-38], but does not report the effect size in the moderately and severely depressed patients. He criticizes the use of exclusion factors, which does reduce the ability to generalize to a broader range of patients, but the exclusion of serious hospitalized depressed or suicidal patients would reduce the generalization to the patients who need and benefit from antidepressants the most. As a result, the effect size of antidepressant might be greater than reported.

Ioannidis also makes a few technical methodological criticisms of clinical trials, which would apply to trials of medical drugs as well, and we agree that these are problem areas for all drugs. We agree with Ioannidis when he notes that drug-placebo difference has decreased in recent years [26,27]. Some of the reasons for this are:

(a) More recent studies exclude suicidal, and the hospitalized more severe depressions, which have a larger drug-placebo difference, but the exact comparison of effect size should not be made due to methodological differences between earlier and more recent trials;

(b) Many of those with low baseline rating scores do not have the type of depression helped by drugs;

(c) Patients who were helped by drugs in the past no longer volunteer for placebo controlled trials;

(d) The more recent trials are not depressed patients of a physician seeking consultation, but rather symptomatic volunteers who answer an ad for a clinical trial, done by the clinical trial companies (working on a contract with pharmaceutical companies) and are paid per case;

(e) The clinical trial companies have difficulty finding patients, and may inflate the baseline rating to ensure that the patient is enrolled in the study, introducing a false improvement in both drug and placebo (baseline inflation has been well documented in recent studies [39-41]);

(f) Volunteers may collect their payment, but not actually take their pills, further reducing drug-placebo differences;

We question his generalization and interpretation of the data to virtually all depressed patients based on data from a limited number of studies of a few antidepressants from mostly mild cases and volunteers, who answer an advertisement.

Psychiatry has undergone a paradigmatic shift in how it conceptualizes depression over the years. An earlier version of DSM (DSM II) viewed mild depression as a psychoneurosis (neurosis with depressed mood), for which psychotherapy was indicated and considered only severe depressions as manic-depressive disease. At that time, the research was just beginning to distinguish bipolar disease from unipolar depression. Also, psychotic depression was not distinguished as a distinct clinical entity from severe non-psychotic depression. Ioannidis states that antidepressants may be useful in a few severe depression cases, but antidepressant monotherapy is not very effective in treatment-resistant depression, severe psychotic depression or bipolar depression, and must combine antidepressants with other types of treatments. The newer DSM III and IV placed milder depression in the category of depression, not neurosis.

We next examined Ioannidis's 6 criticisms suggesting that the biases reduce the effect size to zero, and believe that they generally operate in the opposite direction, and would be expected to improve effect size, if taken into consideration.

i) Studies have outcomes that are "non-relevant outcomes", that is the average rating scales change is too small of an improvement to be clinically relevant:

We use the clinically important definition that to be a responder, a patient must show a 50% percent improvement or greater. His assumption that a numerically small average difference on a rating scale is flawed because all patients did not have exactly the same mean improvement. Some were remitted, even though most were not.

ii) Studies are too short:

The long-term studies, including the meta-analysis he quoted generally showed larger effect sizes than the shorter studies he noted.

iii) The statistics used falsely inflated drug-placebo differences:

Ioannidis says that including patients in the analysis when they dropped out of the study (as with last-observation-carried-forward analyses) "may lead to overestimates of treatment efficacy in some circumstances." The primary reason depressed patients drop out in the placebo arm is that their [42] depression worsened. In many cases, there is concern on the part of the clinician such as the risk of suicide, worsening depression, suffering, and suicidal ideation, leading the clinician for ethical reasons to withdraw the patient from the trial and to initiate non-blinded treatment. Overall, the dropout rate from clinical trials for poor efficacy is 5 times more frequent in the placebo arm [43]. If such patients are eliminated from the analysis as suggested by Ioannidis, an underestimate of drug efficacy will result. Studies using the newer, favored statistical models, recommended instead of last-observation-carried-forward techniques, show greater drug difference, which is the opposite of what Ioannidis asserts [42-47].

iv) Too many exclusion criteria might inflate drug-placebo differences:

It is true that exclusion of patients often reduces generalization, but the exclusion of suicidal and seriously depressed patients reduces drug-placebo differences, which is the opposite of what Ioannidis's concludes.

v) Placebo lead-in periods falsely inflate the drug-placebo differences:

A lead-in period of usually a few days or a week during which time placebo may be given might have the opposite effect of what Ioannidis asserts [35,45-48]. If previous drug treatments are not washed out completely with sufficient lead-in periods, this would make the placebo effect greater, the opposite of Ioannidis suggestion. In any case, eliminating the washout period impacts both the drug and placebo group equally in a double-blind trial, holding the lead-in effect constant for the trial itself. In so far as there is an effect, it seems to be in the opposite directions from that postulated by Ioannidis [49,50].

vi) Use of multiple groups (3, 4, or 5 groups) versus one placebo group is unethical and might reduce drug-placebo difference:

Ioannidis criticizes studies with multiple drug comparisons, but multiple experimental drug arms are generally dose-finding studies. He does not recognize that studies of this type are important to establishing therapeutic dose range of a drug. The use of too low a dose for full efficacy clinically would result in patients being exposed to side effects but without the benefit of efficacy, and the use of too high a dose would expose patients to unnecessary side effects with no greater efficacy. Both issues demand dose ranging clinical trials and satisfy equipoise concerns. These trials are necessary to find the best dose for efficacy while exposing the patient to the least risk of side effects. This is an essential component of balancing the risk-benefit ratio for any medical therapy. Furthermore, most meta-analyses Ioannidis quotes and other recent meta-analysis trials he cites used relatively low treatment doses, with the use of too low a dose resulting in an underestimate of true effect size [1,35-37]. Furthermore, dose-ranging studies empirically have a lower effect size than two-arm studies.

Beyond these issues, one can speculate that antidepressants are even more efficacious than can be documented due to current clinical trial design limitations. For example, many patients may respond to a second drug when the first does not work. We cannot study the full degree to which they shorten an episode for ethical reasons, as this would require keeping patients on placebo for several years.

Ioannidis recognizes that unknown side effects could produce harm but does not recognize that unknown benefit could also occur. An example of this is the benefits from treatment shortening an episode. Let us explain: since it is generally considered unethical not to treat acute depressions with drugs after a placebo period of 4-6 weeks, we have little information about this period of time, but there is good data from a large NIMH-funded treatment-resistant depression trial, the STAR*D trial, that continual treatment produces an improvement rate of 67% whereas the initial antidepressant treatment of these patients produced an improvement rate of about 33%. There is further benefit from prevention of relapse, and from other outcomes not measured in most trials or in any trial. One can speculate that real efficacy is less or more than that which is measured. **Our argument is not that the antidepressants really have greater benefit than reported, but rather, that it is not valid to conclude that they have no efficacy based on speculations without specific evidence and where the existing evidence shows the opposite**.

## Do antidepressants pose substantial clinical risk?

There have been concerns regarding whether certain antidepressants may cause suicides. We now know this is a myth largely fueled by the media [51]. A few placebo-controlled trials reported that antidepressants caused an increase in suicidal ideation, but actually none of the studies found antidepressants increased completed suicides [52-55]. Newer studies of children do not confirm an increase in suicidal ideation; instead, they show that almost all antidepressants actually help suicidal ideation more than placebo. Furthermore, studies of adults found that antidepressants actually decreased the amount of suicidal ideation, suicidal behavior, or suicide itself [56,57]. Naturalistic studies show that the incidence of suicide rate tends to go down as the incidence of antidepressant treatment goes up [54,56,58-64] and in general suggest that antidepressants prevent suicide [65]. Another complication is that depression in children and adolescents is often the first sign of a bipolar illness, a disorder for which different drugs are needed. Additionally, antidepressants may rarely induce akathisia, which itself is a risk factor for suicidal ideation and behavior. Close monitoring by a physician, who is alert to this possibility, is crucial in order to reduce all adverse outcomes, which has a negative impact on efficacy. After the FDA issued a black warning against antidepressants, antidepressant prescriptions for this population diminished and there has been a concomitant increase in actual suicide rates [54,63,66]. Indeed, before biological treatments were discovered, about 5 to 15% of persons with depression died by suicide [6-8] There are treatments that should not be used, even if the patient wants them, a determination based on weighing the evidence for both benefit and risk.

## Economic Perspective

Individuals with depression suffer high rates of short-term work disability and cost employers $44 billion annually in lost productive time [67]. Mental-health services research has shown the benefits of treating depression with respect to family function, general health, and vocational function. There is evidence that more intensive treatment reduces these costs [68-70]. An NIMH supported randomized blinded study of maintenance extension study to 28 months after initial treatment found significant gain of drug over placebo in social and role functioning [71]}. To put drug costs into perspective, note that the cost of fluoxetine (the generic name for Prozac) is $17 per month, or $200 per year. Low cost generics are available as alternatives to many of the more costly-branded antidepressants. The NIMH supported double-blind randomized trial of fluoxetine versus placebo for adolescent depression, found that fluoxetine has an incremental cost of about $1.20/day, but the incremental benefit of drug over placebo for 3 months treatment is estimated to be $23,737.

## Medical Perspective

There is not a one-to-one absolute correspondence between effect size in clinical trials and the real world effectiveness in general medicine, which is a far different and a more complex question that requires a much broader approach, considering all that is known about a drug and disease. Most drugs do not cure all patients. In the first trial of penicillin, when only a small amount of drug had been made, many patients died, but others had improved until the available supply of penicillin was used up. At the time when penicillin was discovered, the sulfa drugs were used, producing 88% recovery, while 12% died. Penicillin reduced the death rate to 6% [72], decreasing the death rate by one-half, an effect size of 0.42. There are studies in which antibiotics are ineffective for certain bacterial conditions [73,74], but it would be a mistake to generalize from this to argue that antibiotics are not effective for life threatening illnesses, just because it is unethical for them to be studied in these clinical situations. Many common drugs used in general medicine have effect sizes much smaller than antidepressants. One could make a similar criticism of the trials most medical treatments and conclude that their efficacy amounts to a myth constructed from 100,000 trials. Surgery is widely used with little support from randomized placebo-controlled trails. It is true that half-dozen psychiatrists have written articles critical of the efficacy of antidepressants and that, historically, mainstream physicians have been wrong about some clinical beliefs. Nevertheless, antidepressants are now the mainstay of biological treatment of depressed patients throughout the world and have been so for about the last 50 years. Ioannidis's recommendations to not use the psychotherapeutic (as inferred from his statement) or pharmacological treatments, or the combination of both treatments, are based on speculations that do not provide a firm basis for abandoning both psychotherapy and pharmacotherapy.

## How Small and Effect Size is Too Small for a Drug to be Used

Ioannidis does not state what amount of drug-placebo difference he would consider clinically significant, but he rejects the drug-placebo differences observed in clinical trials (12-35% depending on type of depression as summarized in Table 1) as not clinically significant. We argue that even 1% of patients who experience substantial clinical improvement represent a benefit, which is particularly meaningful to those individuals. Benefits can accumulate over time, and depression is frequently a recurrent disease. To illustrate the ethical aspect of this issue, we use a hypothetical example of anti-cancer drug treatment where the underlying cancer has a death rate of 10% per year and drug treatment reduces this by 1% per year. This one percent lives-saved would add up and increase the percentage of total survivors. Should we offer the patients this 1% chance of survival? This 1% benefit would accrue year after year to as much as a 10% cure over a person's life span. How small an effect size should be regarded as clinically insignificant?

## Perspectives from the Philosophy of Science and Logic

Empirical science cannot prove anything with absolute certainty. Some label the reasoning that if something can occur, it will occur, and does occur all the time, the fallacy of "appeal to probability." Ioannidis offers no actual evidence that they do reduce antidepressant efficacy to zero; these are just speculations and yet, he concluded that the opposite of the empirical evidence is true. The erroneous logic is that, if you can criticize the evidence, then you can conclude that the opposite of what the evidence shows must be true. This has certain characteristics in common with the Creationist attack on evolution. Creationists argue that evolution cannot completely explain everything; therefore, the opposite of evolution (i.e. intelligent design) is true.

## Practical and Ethical Constraints on Ethical Clinical Trials

There are IRBs and ethical constraints as well as practical limitations to clinical trials, deserving comment in a journal on ethics and philosophy. Ioannidis calls for large, long-term studies (by implication over 5 years) and asks that some of these "should include suicide, and major life events" such as "loss of job." The trials of 50,000 patients for drug plus presumably another 50,000 ill individuals receiving only placebo would result in a trial that the IRBs would not approve. This fictional trial could never be conducted and completed and would represent too great a health risk for the placebo group. Physicians would not generally refer symptomatic depressed patients for whom antidepressants were clinically indicated to such a trial, and not enough patients would volunteer, absent a considerable monetary payment. Moreover, patients randomized to placebo might still withdraw prematurely, due to lack of efficacy of placebo, and thus produce erroneously skewed results. Our argument is not that such a study would yield more information about these outcomes, but rather it cannot be done for ethical and practical reasons. We feel funding should be directed to identifying which patients should receive medication, and many a host of other questions.

## Philosophy of Ethics

The patient voluntarily visits a physician to seek help. The physician may diagnosis "depression," determine that an antidepressant is indicated for that particular patient and then recommend it, based on that patient's history, current status, and other clinical factors. We consider the recommendation of treatment using the four ethical principles of the Belmont Report, refined by Beauchamp and Childers [75] of: (1) beneficence, (2) non-malfeasance, (3) autonomy and (4) justice. Beneficence, the antidepressants' benefit (or the lack thereof), the main focus and our disagreement, is straightforward. Ioannidis does not dwell on the side effects of the antidepressants, but, most of the antidepressants currently used do have clinically significant side effects. Physicians must work with the patient to determine if the benefits of the drug are worth the particular side effects the patient may be exposed to or experience. We agree on avoiding malfeasance from known and unknown drugs' adverse effects. Ioannidis makes no statement about the possible harm resulting if treatment is not given due to suicide, poor daily functioning and adverse family impact. The alleviation of suffering, in our view, substantially outweighs a few side effects. It is important that the physician be attentive to and minimizes side effects. We see no violation of the patient's autonomy in the recommendation to take medication or in the patient's voluntary action to take medication. We recognize that the physician has an interest in treating depression and could over-value treatment as opposed to doing nothing, and feel the physician should guard against this. But by the same token, the researcher has an interest in doing research and may over-value the importance of research. An example from the history of medicine is the case studies of an investigator who failed to treat with penicillin because he felt that a more systematic study of the natural history of syphilis was needed. The fourth ethical principle is justice. Imagine the hypothetical of a yet unborn medical ethicist with full mental capacities and current knowledge but not knowing whether they would suffer from depression. We think they would wish to have antidepressants available, which, upon becoming seriously depressed, they could choose or not choose to take for themselves depending on their own judgment. Another ethical test to consider is does the availability of antidepressants, which physicians may or may not recommend and patients take or not take, produce harm to society in any imaginable way? (An example of this is: widespread lying and not keeping your word would harm society.) Since Ioannidis condemns the use of behavioral and psychological treatments as well as antidepressants, it is a question of choice of one versus the other, as both can be used. There are also societal aspects of justice. Depression is an illness that results in suffering impacting the patients and their significant others such as family, friends, and coworkers. Moreover, poor vocational and social deficits have societal costs. It is not so costly that only the affluent can afford them.

## Legal Perspective

Physicians also have a medical-legal obligation to try to assuage the suffering and restore functioning through interventions. Some have been sued and have lost litigation when ideological reasons perpetuate unnecessary suffering (see Osheroff v. Chestnut Lodge, Inc [76]). Dr. Osheroff was a patient in a facility that offered psychoanalytic psychotherapy for his severe depression for months; when he was finally transferred elsewhere and received antidepressants and antipsychotics, his depression remitted quickly.

## Anthropological Perspective

The underpinnings of cooperation, empathy, and many similar mental functions are fundamental to human society. All cultures have religious and ethical norms to provide help to members of their society, including the relief of suffering. To alleviate human suffering is a moral imperative.

## Summary

Ioannidis suggests the efficacy of antidepressants is a myth and that same applies to behavioral interventions. There is merit in many of his methodological concerns, which apply to most pharmacotherapy, and behavior intervention. We agree that science, like all empirical knowledge is not perfect. It is important to note that failure of drug companies to publish all their studies does result in an inflated estimation of their efficacy. We agree that this is important. However, we disagree that antidepressant have no greater efficacy than placebo. He notes that there have been over a thousand controlled trials, yet he bases his argument on very little data, indeed just a few meta-analyses. We present the efficacy from approximately a hundred of trials in percent of patients with a substantial clinical response (defined as 50% improvement or more symptomatic reduction). These meta-analysis find 42-70% of antidepressant improve with drug and 21%-39% improve with placebo. The increment with drug is 33%-11% greater than placebo. Ioannidis argues that it would be vanishingly smaller because systematic biasing in these clinical trials would reduce the drug-placebo difference to zero. (To reduce to zero, one would also have to postulate that some of the unpublished studies do find placebo better than drug and substantial systematic bias, without any evidence). His argument that effect size vanishes, suffers from failures of logic because Ioannidis does not present any evidence that there are a large number of studies where placebo is substantially more effective than drug. (To reduce to zero, one would also have to postulate that some of the unpublished studies do find placebo to be better than drug). We also present the empirical evidence showing that these methodological issues generally have the opposite effect of what Ioannidis argues, suggesting that the measured efficacy underestimates true efficacy. Our argument is not that antidepressants are more effective than measured, nor that the effect size is necessarily bigger than Ioannidis' effect size of 0.31, **but rather that he presents no credible evidence that antidepressants have zero effect size**. Our most important criticism is the basic underlying argument that if the existing evidence is imperfect and methods can be criticized, then the findings are invariably wrong, proving the opposite of efficacy, i.e. the drug has no efficacy. We note the similarities of this to the creationist argument that evolution cannot explain everything; therefore, one must postulate intelligence, the opposite of evolution, i.e. intelligent design is true. Depression causes great suffering, indeed so great that 5-15% of depressed patients kill themselves [6,7]. Depression leads to suffering of family and friends. We find no ethical reason why therapists should not try to help depressed patients with drugs and/or with psychotherapeutic/behavioral treatments even though they do not help everyone and our knowledge is not perfect. The immense suffering of patients with major depression leads to professional, ethical, and moral obligations to treat patients with the best available tools at our disposal, while diligently and actively monitoring for adverse effects, and actively considering revisions of the treatment components, whatever the modality employed. Medical decisions should be shared decisions made with the patients, respectful of the patients and families experiences, intuitions, values, and autonomy.

## Competing interests

Stefan Leucht received speaker/consultancy/advisory board honoraria from SanofiAventis, BMS, EliLilly, AstraZeneca, Essex Pharma, GlaxoSmithKline Janssen/Johnson and Johnson, Lundbeck, Medavante and Pfizer.

SanofiAventis and EliLilly supported research projects by SL. The other authors have no competing interests.
